# Sensor for Measuring Strain in Textile

**DOI:** 10.3390/s8063719

**Published:** 2008-06-03

**Authors:** Corinne Mattmann, Frank Clemens, Gerhard Tröster

**Affiliations:** 1 Wearable Computing Lab, ETH Zürich, Gloriastrasse 35, 8092 Zürich; E-mails: mattmann@ife.ee.ethz.ch; troester@ife.ee.ethz.ch; 2 EMPA Dübendorf, Überlandstrasse 129, 8600 Dübendorf; E-mail: Frank.Clemens@empa.ch

**Keywords:** strain sensor, textile integrated, elastomer

## Abstract

In this paper a stain sensor to measure large strain (80%) in textiles is presented. It consists of a mixture of 50wt-% thermoplastic elastomer (TPE) and 50wt-% carbon black particles and is fiber-shaped with a diameter of 0.315mm. The attachment of the sensor to the textile is realized using a silicone film. This sensor configuration was characterized using a strain tester and measuring the resistance (extension-retraction cycles): It showed a linear resistance response to strain, a small hysteresis, no ageing effects and a small dependance on the strain velocity. The total mean error caused by all these effects was ±5.5% in strain. Washing several times in a conventional washing machine did not influence the sensor properties. The paper finishes by showing an example application where 21 strain sensors were integrated into a catsuit. With this garment, 27 upper body postures could be recognized with an accuracy of 97%.

## 1. Introduction

There is an increasing trend of integrating intelligence in our daily environment. These systems can provide us with information we need in the current situation and helping us to master everyday life more efficiently. Currently, most of these systems are attached to the body (e.g. heart rate monitoring) or integrated in our environment. However, integrating them into the clothing would have some major advantages as they are personal, comfortable, close to the body, and worn almost anywhere and anytime. Additionally, clothing provides a large surface which can be used for sensing, actuating and integrating processing power.

Measuring strain in textile is a problem addressed by different research groups. One of the first groups who mentioned this problem was Farringdon et al. in 1999 [1]. They built a knitted strain sensor which was integrated into a jacket and was used to measure upper body movements. Gibbs et al. designed a textile potential divider to measure joint movements [2]. In [3] a thin layer of polypyrrole (using chemical vapor deposition) was applied on the fabric substrate at low temperature. With this configuration, a measurement range of up to 50% strain and a strain sensitivity ( $\frac{\Delta R}{\varepsilon R_{0}}$, *ε* is the deformation) of 80 was achieved. Das et al. investigated ethylene-vinyl acetate (EVA) and ethylene-propylenediene rubber (EPDM) composites for sensor applications [4]. Such carbon composite materials show high relaxation behavior and creeping which means that the change in resistivity is influenced by the strain rate. An elastomer/carbon black-composite (CE) was used by Tognetti et al. to measure arm and finger movements. This sensor showed high relaxation behavior too [5–7].

Carbon black/elastomer- and rubber-composites need to be cured after compounding and shaping. In contrast, when using thermoplastic elastomer (TPE) based composites, curing is not necessary and simple thermoplastic processing technology can be used for shaping. Therefore, such polymers are interesting when developing strain sensors with large strain [8–10]. In [10], Cochrane et al. presented a sensor of a thermoplastic elastomer and filled with carbon black (27.6vol-%). They focused on noncyclic strain sensing and looked at influences of temperature and humidity on the resistance. The sensor showed a dependance of the resistance on the humidity but not on the temperature. In this paper we use a similar composition (50wt-%/ 32vol-% carbon black) but focus on the characterization of the sensor's dynamic behavior, as sensors integrated into textiles are exposed to repeated strain cycles.

Integrating strain sensors into textiles opens new applications: For example when attaching the sensors in the knee or elbow region, the bending angle of the joints can be measured. Such a measurement can be used in sports (e.g. to measure the number of steps and the speed while jogging) or in rehabilitation to give the patient an online feedback whether he uses the injured joint in the appropriate range. One could even think of a whole body posture measurement which enables a quality and quantity measurement of exercise conduction in fitness training and rehabilitation. Measuring the posture using strain sensors enables an unobtrusive integration into textile currently not possible with other sensors (e.g. accelerometer, gyroscope, and magnetometer [11]).

In this paper we present a sensor which measures strain in textiles. In Section 2 the sensor material and its textile integration are described. This is followed by an electrical characterization of the sensor: The measurement setup is presented in Section 3, the corresponding results in Section 4. The paper finishes with an example application and a Conclusion.

## 2. Sensor Design

### 2.1. Sensor Material

For the development of the stain sensitive conductive fiber a mixture of a thermoplastic elastomer (TPE) and carbon black particles was used. As described in Section 1, no curing is necessary when using TPE based composites and for shaping simple thermoplastic processing technology can be used. The TPE material was SEBS-Block copolymer THERMOLAST K® (FD-Series), Compound No. TF7-ATL produced by KRAIBURG TPE GmbH, Germany. The carbon black powder was ENSACO 250 produced by TIMCAL, Belgium. The density of the TPE was $0.89\frac{g}{cm^{2}}$ and of the carbon black powder $1.8\pm 0.2\frac{g}{cm^{2}}$. The primary particle size and the specific surface area of the carbon black powder were 54*nm* and $65\pm 5\frac{m^{2}}{g}$ respectively.

For the fibre manufacturing, TPE pellets were filled in an electrically heated torque rheometer (Rheomix 600, Fisher Scientific, Germany) with roller blade configuration. After melting the thermoplastic part of the TPE, carbon black powder was added and subsequently homogenized and dispersed into the polymer during 1 hour at 180°C. The rotation speed was constant (10rpm) during the whole procedure.

After compounding, the fibre was produced by using a capillary rheometer (Rosand RH 7, Malvern Instruments, Germany) and an extrusion die with an orifice of *300μm*. The composite material was preheated and -compacted in the cylinder of the rheometer for 2*min* and a pressure of *0.5MPa* at 180°C before extruding with speed of 3.5*mm/min*. Because of die swelling, the fibre diameter increased to 315*μm*.

**Figure 1.**
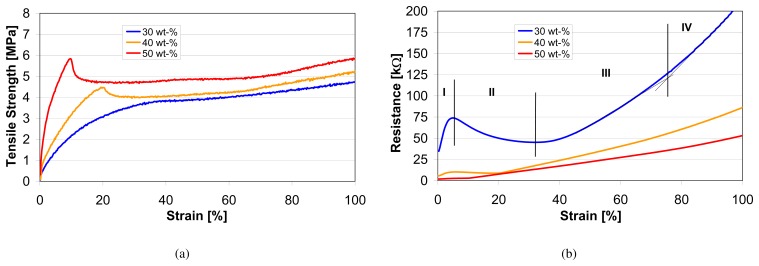
Influence of the carbon black filler content on the mechanical (Fig. 1(a)) and the electrical (Fig. 1(b)) properties of the extruded fibre with a gauge length of 5cm. In Fig. 1(b), the four regions described by Flandin et al. [13] are shown for a filling level of 30wt-%.

In a previous investigation [12], different contents of carbon black were added to the TPE polymer to study the electromechanical properties of the fibres. Fig. 1 shows the influence of the carbon content on the mechanical (tensile strength) and the electrical (resistance) properties of the extruded fibre.

**Figure 2.**
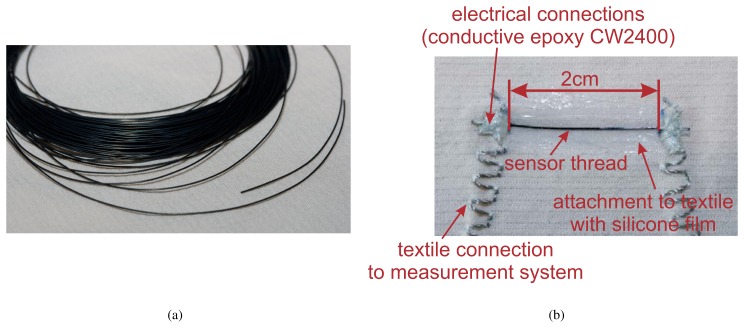
Fig. 2(a): Sensor thread after extrusion. Fig. 2(b): Sensor thread attached to the textile with a silicone film.

As expected, varying the content of carbon black in the compound influences the mechanical and electrical properties. Above a certain amount of carbon black (40wt-%), a yield point occurs in the mechanical stress strain curve of the sensor fibres. The electrical behavior of the fibre changes too. For low filling levels of carbon black (30wt-%) the resistivity curve can be separated into four sections as described by Flandin et al. - initiation (I), reversible (II), re-coverable damage (III) and depercolation (IV) [13]. Such a composite cannot be used for a strain sensor with a large range up to 100% strain as the same resistivity occurs at different strains. Fibres with content around 40wt-% show a plateau in the electrical resistivity (10% - 20%) which changes to a monotonically increasing curve for higher filling levels (50wt-%). A further increasing of the filling level results in a very brittle fibre which is unacceptable for sensor applications. Therefore, a 50 wt-% composite ( $1.21\frac{g}{cm^{2}}$, 32vol-%) was used to produce the textile strain sensors in this study (see Fig. 2(a)). This filling level results in a resistance of approximately 700Ω/*cm*.

### 2.2. Textile Integration

As mentioned in Section 1, the sensor was designed to measure elongations in textiles. Therefore, the sensor thread described in Section 2.1 (see Fig. 2(a)) needs to be integrated or attached to a textile before characterization. This attachment was realized with a silicone film (Dow Corning 732) (see Fig. 2(b)) which enables a measurement range of 100% strain. A silicone film was used for the following reasons:

- Silicone is very elastic. It has the ability to undergo extreme flexing, but still retaining its original shape without any significant loss of its property profile ([14], Flex Life). Additionally, it is resistant to ageing and weathering, providing a good protection to the sensor, and it is inert when being in contact to the skin.
- Silicone builds a good bonding to textiles. Therefore, by using silicone the sensor is reliably attached to the garment.
- When stretching the sensor material described in Section 2.1 alone, it experiences a permanent plastic deformation: The sensor gets longer and does never return to its original length. Therefore, the sensor material alone has a reduced elongation range of the applied stretch minus the permanent deformation. However, when the sensor is attached to the textile using silicone, the sensor is forced back to its original length and, therefore, has a larger working range.

For connecting the sensor to a measurement system, a *SHIELDEX*® yarn (235f34dtex 2-ply HC) was used. This nylon yarn is silver coated and has a resistivity of 120Ω/*m*. In order to keep the elasticity of the textile, the thread was sewn using an elastic stitch. For the connection between the sensor and the silver coated yarn, a conductive epoxy (CW2400, CHEMTRONICS CIRCUITWORKS) was used.

**Figure 3.**
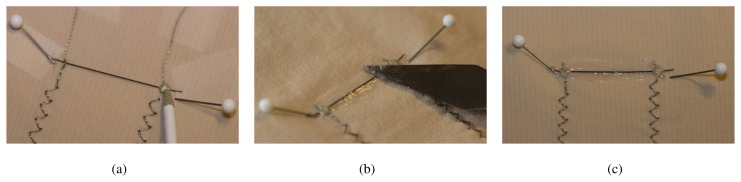
Assembling of the textile strain sensor. Fig. 3(a): Connecting the sensor to the conductive thread using conductive epoxy. Fig. 3(b): Attaching the sensor to the textile using silicone. Fig. 3(c): Sensor attached to the textile.

In Fig. 3 the assembling of the sensor is shown. It comprises the following steps:

1. In a first step, the sensor is temporary attached to the textile using adhesive tape. Thereafter, the textile is attached to a cardboard with fixing pins under light tension in order to prevent shifting when using the conductive glue and to prevent the building of ripples when applying silicone.
2. The sensor is then connected to the conductive thread using conductive epoxy (see Fig. 3(a)). This step needs a drying period of 24 hours.
3. In a next step the sensor is attached to the textile with silicone using a palette-knife (see Fig. 3(b)). The silicone needs some hours to cure.
4. After removing the adhesive tape and the fixing pins, the textile strain sensor is finished (see Fig. 3(c)).

## 3. Measurement Setup

Several cyclic strain measurements (extension - retraction) were performed in order to characterize the sensor. For these measurements, the sensor thread was attached to the textile as described in Section 2.2. The sensor length was always 2*cm*. This length will be used in a typical application.

All measurements were performed with a strain tester (Zwick/Roell DO-FB0.5TS). The resistance was measured in parallel with a multimeter HP34401. If not specified differently, the measurements were done at a speed of 200*mm/min* which corresponds to a strain rate of 16%/sec (sensor length 2*cm*) which is achieved in typical body movements. The waiting times at maximal and minimal strain were 3*sec*.

The following measurements were taken (For each of these measurements, a different sensor was used.):

**increasing range** The measurement range was slowly increased (steps of 10%) starting with a range of 10% up to a range of 100% strain. With this experiment, some insight can be gained on the influence of the measurement range on the sensor characteristics.
**decreasing range** Starting with a measurement range of 80% strain, the range was decreased in steps of 20%. This measurement should confirm that the characteristic curve (= strain-resistivity characteristics) only depends on the maximal strain applied and remains constant for arbitrary (smaller) strains.
**varying speed** The strain rate was varied between 50*mm/min* and 600*mm/min* (50, 100, 200, 400, and 600*mm/min)*, covering a typical motion speed range of the human body. This measurement gives information about an influence of the strain rate on the sensor characteristics.
**waiting times of 2 minutes** The waiting times at maximal and minimal strain was increased to 2*min* in order to see the relaxation behavior of the sensor.
**long-term cycling (16 hours)** The sensor was permanently cycled for 16 hours. This shows whether there is a change in the characteristics of the sensor while in permanent use.
**long-term measurement (2 months)** During two months, the characteristics of the sensor was measured once a week in order to find ageing effects of the sensor.
**washing test** The sensor was washed 8 times at 30°C in a washing machine using a conventional cleaning agent (no fabric softener). After each washing cycle, the characteristics of the sensor was measured. This trial shows whether the sensor endures this procedure and the properties change due to washing.

All measurements were done with the sensor attached to two different textiles (486 Meryl (88% PA, 12% lycra, knitted) and Keller AG 88018 (49% PA, 51% EL, woven)) which have different elasticities. The Meryl textile is about three times more elastic than the weave from Keller AG. However, the sensor characteristics were the same so that the kind of textile is not considered in the following sections.

## 4. Results and Discussion

In the following section, the textile integrated sensor thread is characterized. The following sensor properties were examined: relaxation behavior, hysteresis, working range, dependency on strain rate, longterm cycling, ageing, and washability. We did not look at influences by temperature and humidity as this is covered in [10] where a similar sensor was used. They found a dependance of the resistance on the humidity but not on the temperature. As our sensor is embedded and, therefore, protected with a silicone film, we expect the influence of the humidity to be reduced.

### 4.1. Relaxation Behavior

**Figure 4.**
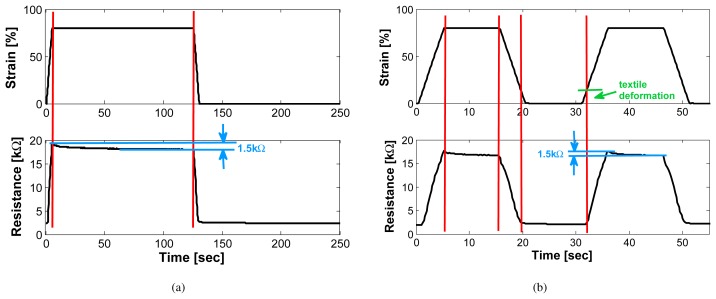
Typical response of sensor to a given strain (sensor length 2cm). Fig. 4(a): waiting time 2 minutes. Fig. 4(b): waiting time 10 seconds

In Fig. 4(a), a typical resistance vs. time plot is shown. The upper plot depicts the applied strain. The sensor was cycled between 0% and 80% strain at a speed of 200*mm/min* and waiting times at minimal and maximal strain of 2 minutes. In the lower plot the measured resistance is shown which varied between 2*k*Ω and 19*k*Ω (for a sensor length of 2cm).

It is apparent that this sensor has a remarkably small creep compared to other textile sensors. When the strain is kept constant, it relaxes by 1.5*k*Ω (see Fig. 4(a)) while the total range is 17*k*Ω. This results in an inaccuracy of 8.8% caused by the relaxation.

For strains lower than 10% it can be seen in Fig. 4(b) that the resistance does not follow the applied strain. It stays at the resistance level which corresponds to a strain of about 10%. This is caused by a temporary deformation of the textile due to the large strain applied (marked as ‟textile deformation” in the Figure). This is not a problem for our application as the garment is pre-stretched when worn and we, therefore, use the sensor in the working range only.

The sensor has a high sensitivity $\frac{\delta R}{\delta l}$ of $1.25\frac{k\Omega }{\text{mm}}(=250\frac{\Omega }{\%\text{strain}})$ and a gauge factor of ∼20 at a sensor length of 2cm.

### 4.2. Hysteresis

**Figure 5.**
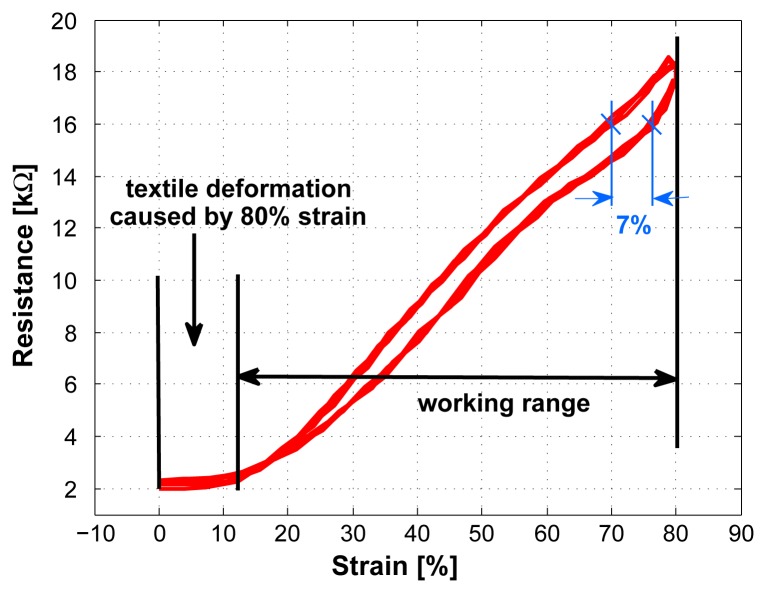
Typical response of sensor to a given strain (sensor length 2cm).

A typical resistance vs. strain plot is shown in Fig. 5, indicating a linear rise in resistance when applying strain and only a small hysteresis. A system with hysteresis is defined as a system whose output does not only depend on the current input but also on the history of the input. Typical causes for hysteresis are friction and structural changes in the material [15].

For the sensor, the maximal hysteresis error is ±3.5% (7%) in strain at 16*k*Ω. The mean hysteresis error over the working range is ±2.25% (4.5%) in strain.

The temporary deformation of the textile, which was described in Section 4.1, can also be seen in Fig. 5. In this plot it appears as a plateau at low strains.

### 4.3. Working Range

In this Section it is analyzed whether the sensor properties remain stable when the sensor is used in different ranges. Two tests were conducted where the strain was gradually decreased and increased, respectively.

**Figure 6.**
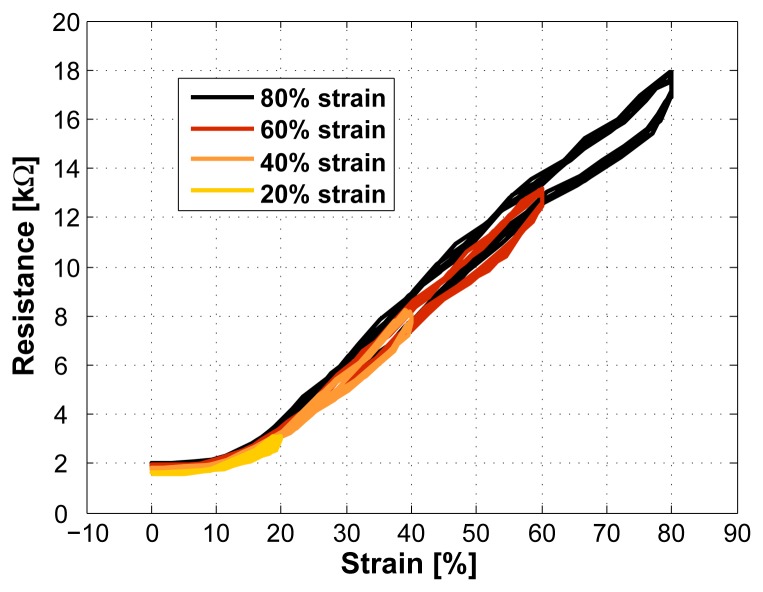
Applying a strain of 80% to the sensor (black curve) followed by a decreasing strain (sensor length 2cm). The sensor characteristics remains constant. We call this the pre-stretched case, which means that the sensor is maximally stretched before any other (smaller) strains are applied.

In Fig. 6, the sensor was first stretched to 80% (black curve). After that the strain was gradually decreased in steps of 20%. This figure shows that the sensor characteristics (slope) remains the same also for smaller strains.

**Figure 7.**
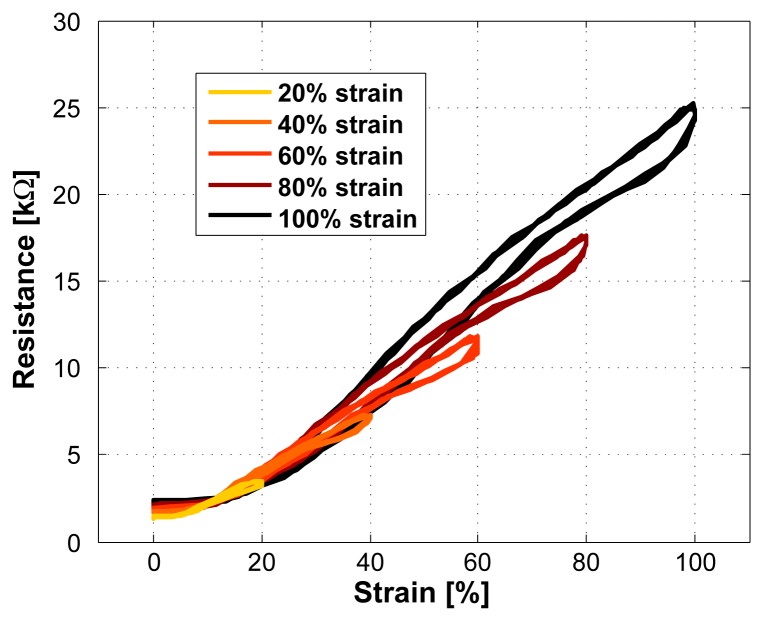
Applying an increasing strain to the sensor without pre-stretching it (sensor length 2cm). The sensor characteristics changes.

In Fig. 7, an increasing strain was applied to the sensor. It can be seen that the sensor characteristics changes with each increase in strain. With such a behavior, the TPE/carbon black composite can only be used as a strain sensor with reduced accuracy. Therefore, the sensor needs to be stretched to its full working range before its first usage so that the sensor properties become stable. We call this a pre-stretching. This also means that during usage, no larger strain than the pre-stretch value should be applied.

To confirm that a pre-stretched sensor remains stable, the increasing strain test in Fig. 7 was repeated with a pre-stretched sensor (the time between pre-stretching and the test was 24 hours). The result was the same behavior as shown in Fig. 6 which confirms that a pre-stretching is necessary and that it ensures stable sensor properties.

While doing the measurements, it turned out that the sensor in the described setup works reliable up to 80% (no failures). When applying a strain of 100% some failures occurred. However, some sensors worked up to much larger strain (150%). Breaks usually appeared while stretching the sensor for the first time. Once stretched to a certain amount without breaking, the sensor worked reliable. Therefore, we specify the maximal range of the sensor to be between 0% and 80% strain.

Two types of failures appeared during the measurements: The sensor either failed due to a rupture of the sensor thread at one of the ends of the sensor (at the transition to the epoxy) or as cracks in the conductive epoxy so that the connection between the sensor and the conductive thread failed.

### 4.4. Dependency on Strain Rate

**Figure 8.**
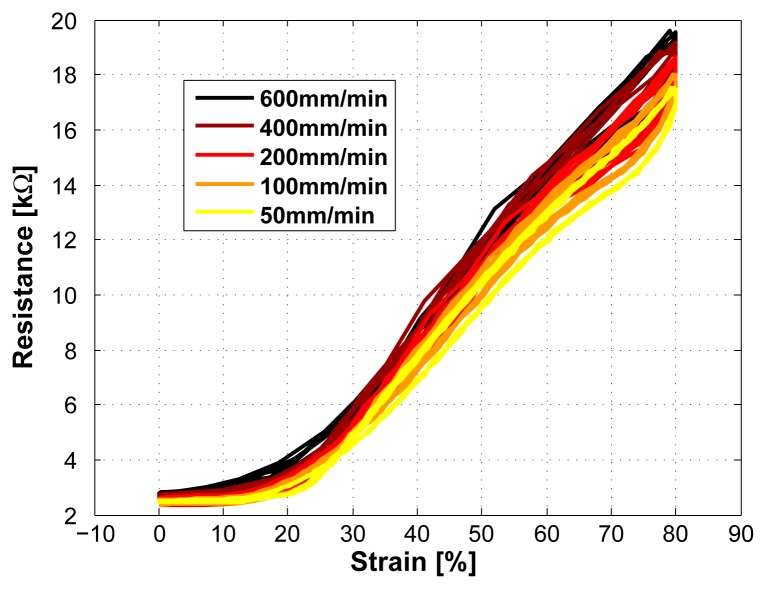
Sensor characteristics at different measurement speeds (50, 100, 200, 400, and 600*mm/min*, sensor length 2cm), covering a typical motion speed range of the human body.

The dependency on the strain rate is shown in Fig. 8. The strain velocity was increased from 50*mm/min* to 600*mm/min* (50, 100, 200, 400, and 600*mm/min*). This increase in speed has shown a marginal rise in resistance. The maximal error increased from ±3.5% to ±7.5% when doing a linear approximation over all five measured speeds and using a range of 80% strain. The mean error increased from ±2.25% to ±5.5%.

### 4.5. Longterm Cycling

**Figure 9.**
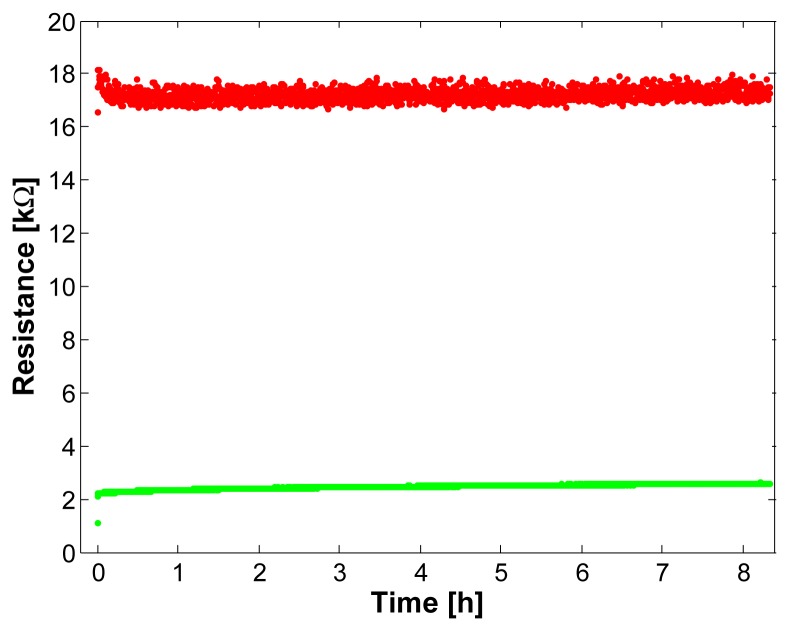
The sensor was cycled for 16 hours whereas the maximum and minimum resistance values of the first 8 hours are shown.

When using the sensor for a longer period of time without any rest, the sensor properties need to be stable. Therefore, a test was conducted where the sensor was cycled for 16 hours at a cycling rate of $\sim 475\frac{\text{cycles}}{\text{hour}}$, resulting in a total of ∼3800 cycles. The maximum and minimum resistance values of the first 8 hours are shown in Fig. 9. It can be seen that the minimal resistance shows a marginal increase of about 0.5*k*Ω. This increase is probably caused by a slowly increasing textile deformation (see Section 4.1). Also the first step at the beginning is caused by textile deformation, however, when the sensor is pre-stretched as described in Section 4.3, this step is much smaller. The maximal resistance decreases in the first half an hour by 1*k*Ω (6%). This phase is followed by a slow increase of the maximal resistance approaching the initial level. Therefore, the sensor properties can be assumed to be widely constant with time.

### 4.6. Ageing

**Figure 10.**
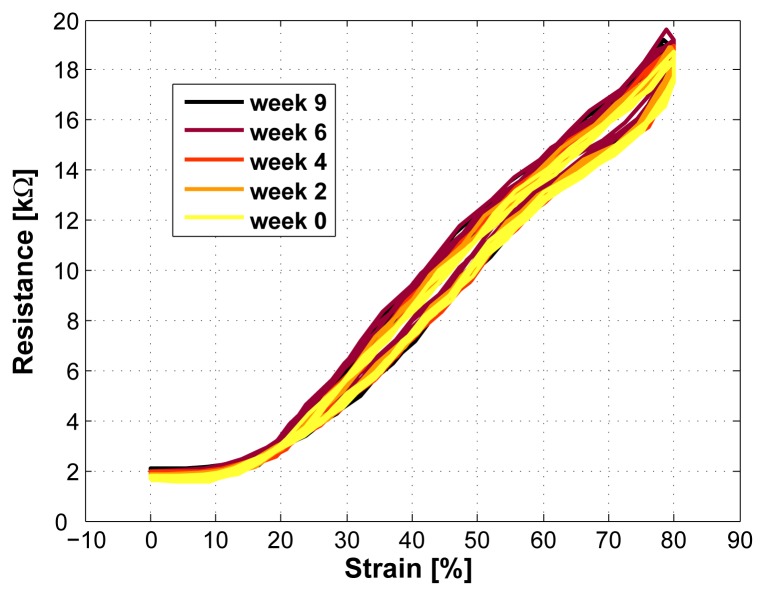
Repeated measurements during 2 months (one measurement per week, sensor length 2cm, measurement speed 200mm/min), confirming the long-term stability of the sen-sor.

In order to show the longterm-stability of the sensor signal, the measurements were repeated once a week during two months. During this time, no drift in the sensor signal was found (see Fig. 10).

### 4.7. Washability

The strain sensor was washed 8 times (over a period of 2 months) at 30°C in a washing machine using a conventional cleaning agent. This had no influence on the sensor properties. One reason for this durability is the attachment of the sensor using silicone. This protects the sensor against many factors.

## 5. Example Application

**Figure 12.**
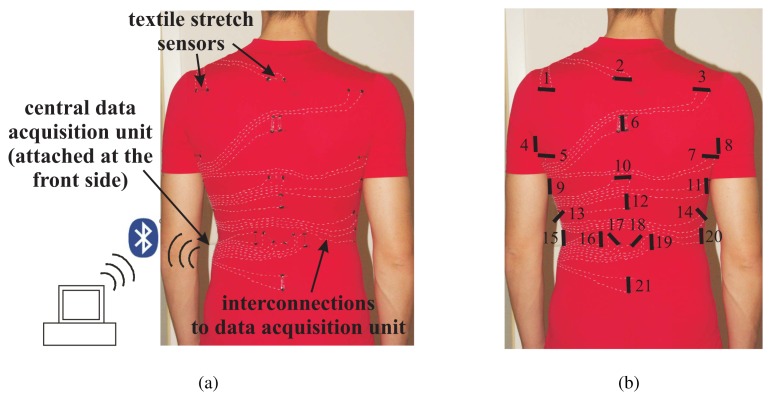
Architecture overview of a prototype recognizing upper body postures using strain sensors (Fig. 12(a)). In Fig. 12(b) the exact positioning of the sensors is shown.

In order to show the functioning of the textile strain sensor, a prototype was built which is able to measure upper body postures [16]. 21 strain sensors were attached to the back region of a tight-fitting clothing (see Fig. 12). With this setup, the sensors measure strain in the garment caused by different body movements and enable to distinguish between a pre-defined set of body postures.

The concept of this prototype was proved in a study with eight participants who performed 27 upper body postures. The participants were instructed to assume the different postures for approx. two seconds while wearing the garment prototype. A picture was shown to the participants to indicate each posture, however, postures were not explained or trained beforehand.


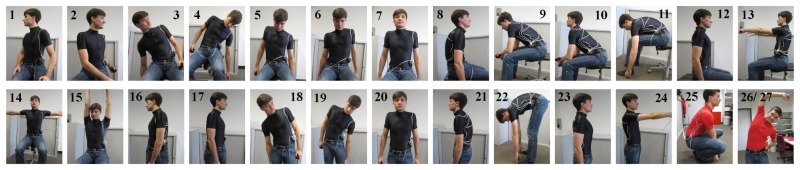


Nearly a complete recognition rate of 97% was achieved with a user dependant training [16]. For an unseen user, a classification rate of 65% was obtained. Hence a posture classification is feasible even for a new user setting. Two types of error were evident in the unseen user classification mode: 1) Confusions appeared between the same sitting and standing postures and 2) similar postures could not be perfectly differentiated.

With this experiment, we could show the feasibility of measuring strain in textiles using the sensor described in Section 2.

## 6. Conclusions

In this paper, we have presented a strain sensor which can measure strain of at least 80%. Commer-cially available strain gauges reach a higher linearity but at a very reduced working range of less than 1%. However, such small measurement ranges do not work for textile integrated applications. The large strain range of the presented sensor opens the possibility to use the sensor to measure strains in textiles. Due to its fiber-shaped form (diameter of 0.315mm) this sensor has the potential to be fully integrated into textiles.

In this paper, different sensor properties were characterized which are summarized in the following.

These properties qualify the sensor for precise strain measurement in a garment.

- linear resistance vs. strain characteristic over a working range of 80% strain,
- a small hysteresis and a minor dependance on the strain rate resulting in a total mean error of ±5.5% in strain,
- stable sensor properties while in continuous usage,
- no ageing effect,
- a high sensitivity of 1.25*k*Ω/*mm* (sensor length 2cm) and a gauge factor of ∼20,
- thread-like shape,
- washable.

Before usage, the sensor needs to be pre-stretched in order to get stable properties.

## Figures and Tables

**Figure 11.**
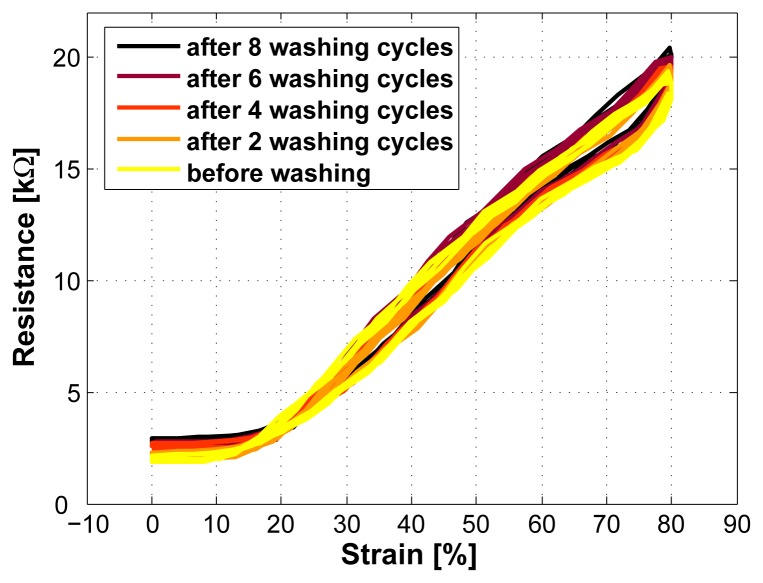
The sensor properties remained stable also after 8 washing cycles.
